# Targeting CXCR4 abrogates resistance to trastuzumab by blocking cell cycle progression and synergizes with docetaxel in breast cancer treatment

**DOI:** 10.21203/rs.3.rs-2388864/v1

**Published:** 2023-02-14

**Authors:** Shuying Liu, Shelly M. Xie, Wenbin Liu, Mihai Gagea, Ariella B. Hanker, Nguyen Nguyen, Akshara Singareeka Raghavendra, Gloria Yang-Kolodji, Fuliang Chu, Sattva S. Neelapu, Samir Hanash, Johann Zimmermann, Carlos L. Arteaga, Debasish Tripathy

**Affiliations:** The University of Texas MD Anderson Cancer Center; The University of Texas MD Anderson Cancer Center; The University of Texas MD Anderson Cancer Center; The University of Texas MD Anderson Cancer Center; University of Texas Southwestern Medical Center; The University of Texas MD Anderson Cancer Center; The University of Texas MD Anderson Cancer Center; University of South California; The University of Texas MD Anderson Cancer Center; The University of Texas MD Anderson Cancer Center; The University of Texas MD Anderson Cancer Center; Spexis Ltd; University of Texas Southwestern Medical Center; University of Texas Southwestern Medical Center

**Keywords:** breast cancer, HER2, drug resistance, CXCR4, trastuzumab, docetaxel, targeted therapy, combined therapy

## Abstract

**Background:**

Although trastuzumab and other HER2-targeted therapies have significantly improved survival in patients with HER2 overexpressed or amplified (HER2+) breast cancer, a significant proportion of patients do not respond or eventually develop clinical resistance. Strategies to reverse trastuzumab resistance remain a high clinical priority. We were the first to report the role of CXCR4 in trastuzumab resistance. The present study aims to explore the therapeutic potential of targeting CXCR4 and better understand the associated mechanisms.

**Methods:**

Immunofluorescent staining, confocal microscopy analysis, and immunoblotting were used to analyze CXCR4 expression. BrdU incorporation assays and flow cytometry were used to analyze dynamic CXCR4expression. Three-dimensional co-culture (tumor cells/ breast cancer-associated fibroblasts / human peripheral blood mononuclear cells) or antibody-dependent cellular cytotoxicity assay was used to mimic human tumor microenvironment, which is necessary for testing therapeutic effect of CXCR4 inhibitor or trastuzumab. The FDA-approved CXCR4 antagonist AMD3100, trastuzumab, and docetaxel chemotherapy were used to evaluate therapeutic efficacy in vitro and in vivo. Reverse phase protein array and immunoblotting were used to discern the associated molecular mechanisms.

**Results:**

Using multiple cell lines and patient breast cancer samples we confirmed CXCR4 drives trastuzumab resistance in HER2+ breast cancer and further demonstrated that the increased CXCR4 expression in trastuzumab-resistant cells is associated with cell cycle progression with a peak in the G2/M phases. Blocking CXCR4 with AMD3100 inhibits cell proliferation by downregulating mediators of G2-M transition, leading to G2/M arrest and abnormal mitosis. Using multiple trastuzumab-resistant cell lines and an in vivo established trastuzumab-resistant xenograft mouse model, we demonstrated that targeting CXCR4 with AMD3100 suppresses tumor growth in vitro and in vivo, and synergizes with docetaxel.

**Conclusions:**

Our findings support CXCR4 as a novel therapeutic target and a predictive biomarker for trastuzumab resistance in HER2+ breast cancer.

## Background

Amplification of the human epidermal growth factor receptor 2 (HER2) /neu (ERBB2) gene and overexpression of the oncoprotein HER2 occurs in around 20% of breast cancers, termed “HER2-positive (HER2+) breast cancer”[1-3]. The humanized anti-HER2 monoclonal antibody, trastuzumab (Herceptin), was the first oncogene-targeted therapy [4], and its use over the last 25 years has improved disease-free and overall survival in patients with early and advanced-stage HER2 + breast cancer [5-8]. However, resistance to trastuzumab remains a clinical challenge; many patients with advanced breast cancers do not respond or eventually develop clinical resistance. Reported underlying mechanisms of trastuzumab resistance include activation of the PI3K pathway [9], upregulation of the insulin-like growth factor-I receptor (IGF-1R) or an increase in IGF-1R/HER2 heterodimers [10, 11], and upregulation of epidermal growth factor receptor (EGFR), HER3/4, or their ligands [12-14], given that trastuzumab is unable to block ligand-induced EGFR/HER2 and HER2/HER3 heterodimers [15, 16]. In addition, variable HER2 C-terminal fragments are kinase-active but lack the trastuzumab-binding epitope [17]. Although many molecules/pathways have been implicated in trastuzumab resistance, the associated mechanisms remain unclear, and no biomarker can reliably predict a lack of benefit from trastuzumab. While several newer therapies have been approved for progressive HER2 + breast cancer, all rely on targeting HER2. Thus, further understanding the underlying mechanism of resistance to targeting HER2 is critical to improve outcomes for this breast cancer subtype.

In our previous studies, by establishing and using trastuzumab-resistant breast cancer cell models, we initially found upregulation of C-X-C motif chemokine receptor 4 (CXCR4), a G protein–coupled receptor of stromal cell–derived factor-1 (SDF-1; CXCL12) in trastuzumab-resistant breast cancer. Knockdown of CXCR4 with shRNA sensitized the cells to trastuzumab [18-20]. Consistent with our findings, a very recent clinical study showed increased CXCR4 expression in trastuzumab-resistant breast cancer tissues and was associated with a higher risk of recurrence [21]. The CXCR4/SDF-1 axis regulates the trafficking and homeostasis of immune cells and hematopoietic stem cells (HSCs) in bone marrow [22-24]. CXCR4 antagonist AMD3100 (plerixafor, Mozobil) is approved for use to mobilize HSCs to the peripheral blood for autologous transplantation [25]. In recent years, upregulation of CXCR4 has been found in many types of solid tumors [26-29]. CXCR4 signaling contributes to cancer metastasis [30-32] and suppresses antitumor immunity [33]. Given the impact of CXCR4 signaling on both tumor behavior and the immune system, its modulation may have cancer therapeutic implications.

In the present study, based on our previous findings of CXCR4 in trastuzumab resistance, we investigated the therapeutic potential of targeting CXCR4 in trastuzumab-resistant breast cancer models, and explored the associated mechanisms.

## Materials And Methods

### Drugs and reagents

AMD3100 and cisplatin were purchased from Selleckchem (Houston TX); trastuzumab, docetaxel and carboplatin from MD Anderson Cancer Center Pharmacy; and recombinant human CXCL12/SDF-1α from R&D Systems (Minneapolis, MN).

### Cell culture and generation of stable cell lines

BT474 and SKBR3 cell lines were obtained from the American Type Culture Collection (Manassas, VA). All other cell lines were from the MDACC Characterized Cell Line Core at The University of Texas MD Anderson Cancer Center. Mycoplasma contamination and identity verification with short tandem repeat were performed regularly by the Core. BT474 and its derived cells were grown in Dulbecco modified Eagle medium/nutrient mixture/F-12 supplemented with 10% fetal bovine serum (FBS). SKBR3 and its derived cells were maintained in modified McCoy 5α, containing 1.5mM L-glutamine, 2200 mg/L sodium bicarbonate, and 10% FBS. All other cells were cultured at Roswell Park Memorial Institute with 10% FBS. For establishment of trastuzumab-resistant cell lines, BT474 and SKBR3 cells were continuously exposed to trastuzumab (20 μg/ml) for at least 1 year. For generation of the cell lines stably knocking down CXCR4, cells were transduced with CXCR4 human shRNA lentiviral particles containing human short hairpin RNAs (shRNA; TL313630VC and TL313630VD; OriGene, Rockville, MD) [34, 35] according to the manufacturer’s protocol. Lentiviral particles containing noneffective scrambled shRNA provided by the manufacturer were used as a control. After transduction for 1 day, cells were selected with puromycin (1 μg/ml) for 2 weeks and pooled.

### Cell growth inhibition assay

Cell growth inhibition assays were performed in three-dimensional (3D) culture in Matrigel as previously described [36]. Briefly, 4×10^3^ cells were re-suspended in growth medium containing 2% growth factor–reduced Matrigel (BD Biosciences, NJ) and seeded in 8-well chambers coated with Matrigel (BD Biosciences). Drugs with SDF-1α (4 ng/ml) were added on day 5. The concentration of SDF-1α was used based on the circulating SDF-1α level in breast cancer patients. Medium with drugs was replaced every 3 days. Acini were photographed and counted in 10 randomly chosen fields. Total number and area of acini were quantitatively analyzed using AlphaView SA software (Cell Biosciences) and expressed as means ± standard deviation (SD), representative of three independent experiments.

### Clonogenic assay

Clonogenic assays were carried out as previously described {Liu, 2019 #32}(36). Briefly, 700 cells were seeded in each well of 6-well plates in the growth medium for 14 days. For inhibitory assays, after attaching to the plate, the cells were treated with drugs for 2 days. Then, the drugs were washed away, and the cells were allowed to grow in the growth medium for 18 days (HCC202, HCC1419, or their derived cells) or 14 days (BTRT and SKRT cells). After staining with 0.25% crystal violet in 20% ethanol, the total number and size of colonies were quantitatively analyzed using AlphaView SA software. Data were expressed as mean ± SD of triplicates, and results were representative of two independent experiments.

### Cell co-culture in 3D

Tumor cells, human breast cancer-associated fibroblasts (BCAF; Neuromics, MN) and human peripheral blood mononuclear cells (PBMC; Zen-Bio, CA) in 3D co-culture were performed as illustrated in Fig. S1 or Fig. 2B. Spheres were photographed at the indicated time. At the end of the study, cell viability was quantitatively analyzed using the CellTiter-Glo 3D viability assay kit (Promega) following the manufacturer’s instructions. Relative luminescence units were measured using a microplate reader. Data were expressed as mean ± SD of triplicates, and results were representative of two independent experiments.

### Antibody-dependent cellular cytotoxicity (ADCC) assay

ADCC assays were performed as described previously [37]. Briefly, human PBMCs were thawed following the protocol provided by the manufacturer. HCC1419-derived tumor cells were harvested and labeled with 5- (and 6)-carboxyfluorescein diacetate, succinimidyl ester (CFDA, SE; Molecular Probes, Inc). After washing, the labeled target cells were mixed with PBMCs at an effector: target ratio of 80:1. Trastuzumab was added to the mixed suspensions at a concentration of 100 μg/ml and incubated at 37°C in a humidified 5% CO_2_ incubator. Ten hours later, the dead cells were stained with propidium iodide and analyzed using a Beckman Coulter Gallios flow cytometer.

### BrdU incorporation assay and dynamic CXCR4 detection

BTRT cells were seeded in the growth medium. After attaching, cells were serum starved for 24 hours then FITC-BrdU pulse–labeled for 1 hour. After washing, cells were cultured in growth medium until collection. For testing the effects of treatment with docetaxel on CXCR4 expression, cells were treated with docetaxel (5nM) for 24 hours before BrdU pulse and after BrdU pulse until cell collection. BrdU was detected with FITC-conjugated anti-BrdU antibody. CXCR4 was detected with the anti-human CXCR4 antibody MAB172 (R&D, Minneapolis, MN), with the IgG_2_B isotype (R&D) as a control, followed by staining with APC-conjugated secondary antibody (Invitrogen). Then cells were counterstained with 7-amino-actinomycin D (7-AAD) and analyzed using Beckman Coulter Gallios flow cytometer with Kaluza Analysis software.

### Cell cycle distribution and Annexin V apoptosis assays

For cell cycle distribution, harvested and fixed cells were treated with RNase A and stained with propidium iodide, followed by analysis using Beckman Coulter Gallios flow cytometer. For apoptotic cell detection, a FITC Annexin V Apoptosis Detection Kit I (BD Pharmingen) was used following the manufacturer's instructions. In brief, cells were treated with AMD3100 (5μM) for 3 days. The cells were harvested and stained with FITC-labeled Annexin V and propidium iodide (PI), followed by flow cytometry analysis.

### Immunofluorescent staining and confocal microscopy analysis

For comparing CXCR4 expression in acquired trastuzumab-resistant cells or their parental cells, the cells were grown on coverslips pre-coated with polylysine and fixed in 4% paraformaldehyde for immunofluorescent staining. For testing the effect of AMD3100 on CXCR4 translocation induced by SDF-1α, the trastuzumab-resistant cells were treated with AMD3100 for 48 hours. After serum starvation overnight, the cells received SDF-1α stimulation for the designed time. For immunofluorescent staining, cells were fixed in 4% paraformaldehyde at room temperature for 20 minutes and permeabilized in 0.25% Triton X-100 for 5 minutes. After blocking with 3% bovine serum albumin for 1 hour, cells were incubated with the CXCR4 antibody overnight at 4°C, followed by staining with Alexa Fluor 488–conjugated goat anti-mouse secondary antibody (Invitrogen). Nuclei were stained with 4’, 6-diamidino-2-phenylindole (DAPI; Thermo Scientific). After mounting, microscopic images were captured by a multiphoton confocal laser-scanning microscope (Carl Zeiss, Thornwood, NY).

### Enzyme-linked immunosorbent assay (ELISA)

Serum SDF-1α from breast cancer patients or similar-aged healthy women was quantified by ELISA using the Human CXCL12/SDF-1α Quantikine ELISA Kit (R&D Systems). Concentrations were calculated by comparing the sample absorbance to standard curves.

### Reverse-Phase protein array (RPPA)

Cells were seeded in 3D Matrigel and treated with AMD3100 (5μM) and/or trastuzumab (20 μg/ml) starting on day 6 for 5 days. The cells were harvested from the Matrigel with pre-cooled 1X HBSS with 5 mM EDTA on ice and lysed in ice-cold lysis buffer [38]. The cell lysates were analyzed with RPPA [39, 40]. The antibodies used are listed in Table S1. Human fresh-frozen tumor tissues were lysed in cold lysis buffer with homogenization and analyzed by RPPA [41].

### Western blot analysis

Cell lysis was prepared as described in the RPPA subsection of the Materials and Methods section. Western blot analysis was performed as described previously [36]. Quantitative analysis of the bands was performed using AlphaView SA software.

### Establishment of trastuzumab-resistant xenograft model and studies in vivo

Five-week-old female athymic nude mice (The Jackson Laboratory, Bar Harbor, ME) were implanted with 0.36-mg, 90-day release 17 -estradiol pellets (Innovative Research, Sarasota, FL). Three days later, 5×10^6^ HR6 cells [12] in 150 μl growth factor-reduced Matrigel and phosphate-buffered saline (1:1) were orthotopically injected. Once tumors reached a volume of ~100 mm^3^, the mice were randomly grouped and received treatment with vehicle, trastuzumab (20 mg/kg, intraperitoneally twice per week), AMD3100 (5 mg/kg, intraperitoneally twice per week), docetaxel (10 mg/kg, intraperitoneally once per week), or combinations as indicated. Tumor sizes were measured with calipers twice weekly. Tumor volume was calculated using the formula V = *lw*^2^/2. Differences in tumor volume between groups were analyzed using two-way ANOVA. At the end of the experiment, the mice were sacrificed with CO_2_. The tumors were harvested and subjected to double-blind histopathologic analysis by a veterinary pathologist.

### Human samples

Tumor and blood samples from breast cancer patients and healthy blood samples along with clinical data were obtained under protocols approved by the institutional review board at The University of Texas MD Anderson Cancer Center. Patients and tumor characteristics were collected by chart review [41]. The Institutional Review Board of MD Anderson approved the laboratory study. The tissues and serum samples were stored at 80°C until further analysis.

### Statistical analyses

One-way ANOVA was used for multiple groups, and the *t*-test was used for two groups. Tumor growth curves were analyzed using two-way ANOVA, using Prism (GraphPad Software, La Jolla, CA).RPPA data were analyzed as previously described [39, 40] and followed by further analysis with one-way ANOVA to compare different groups. Data were expressed as mean ± SD. P values less than 0.05 were considered statistically significant.

## Results

### CXCR4 drives primary trastuzumab resistance in HER2+ breast cancer, and pharmacologic inhibition of CXCR4 sensitizes the cells to trastuzumab

**Figure 1:**
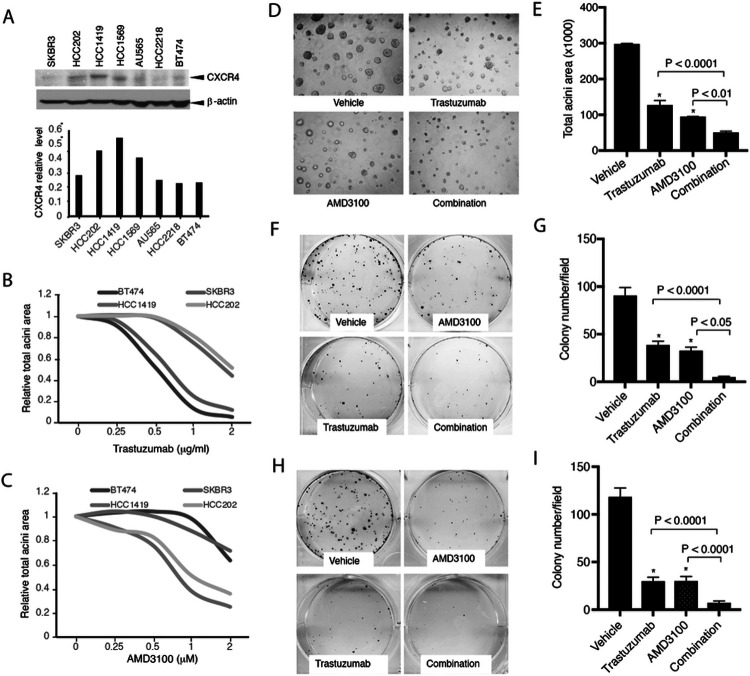
Targeting CXCR4 abrogates trastuzumab resistance. **A** HER2+ breast cancer cells with or without primary trastuzumab resistance were examined for CXCR4 expression with its antibody (UMB2, Abcam) using Western blot analysis. The density of the bands was quantitatively analyzed. **B, C** Cell lines with high expression of CXCR4 (HCC1419 and HCC202) and low expression of CXCR4 (BT474 and SKBR3) were seeded in 3D Matrigel and treated with trastuzumab (**B**) or AMD3100 (**C**). The total area of the acini was quantitatively analyzed (see Materials and Methods). **D** HCC1419 cells grown in 3D Matrigel culture were treated with trastuzumab (2 μg/ml), AMD3100 (1μM), or the combination. Photographs were taken on day 13 after the start of treatment. The total area of the acini was quantitatively analyzed using AlphaView SA software **(E). F-I**Clonogenic assay. HCC1419 (**F**) and HCC202 (**H**) cells were seeded at low density and treated with AMD3100 (0.5μM), trastuzumab (2.5μg/ml), or the combination. The plates were scanned on day 18 after the start of treatment. Colony formation was quantitatively analyzed using AlphaView SA software. **E, G, I** Data were analyzed using one-way ANOVA and are reported as mean ± SD of triplicates, representing two independent experiments (*P < 0.0001 compared with vehicle).

To confirm CXCR4 contributes to trastuzumab resistance, we analyzed CXCR4 protein expression in multiple HER2+ human breast cancer cell lines that were confirmed with different sensitivities to trastuzumab [42]. Compared with the trastuzumab-sensitive cell lines, the trastuzumab-resistant cell lines exhibited higher CXCR4 expression (Fig. 1A). To investigate the functional role of CXCR4, we used cell lines with high CXCR4 expression (CXCR4-high; HCC1419, HCC202) and low CXCR4 expression (CXCR4-low; BT474, SKBR3) for further studies. Cells were treated with serial concentrations of trastuzumab in 3D Matrigel culture. CXCR4-high cells showed higher tolerance to trastuzumab than CXCR4-low cells (Fig. 1B). Trastuzumab-resistant cells exhibited more sensitivity to the CXCR4 antagonist AMD3100 (Fig. 1C). The combination of AMD3100 and trastuzumab in CXCR4-high HCC1419 cells significantly increased the inhibitory effects on acini growth than either monotherapy (P < 0.0001 compared with trastuzumab alone, P < 0.01 compared with AMD3100 alone; Fig. 1D and E). We also investigated the role of CXCR4 in cell survival using clonogenic assays. AMD3100 or trastuzumab each individually inhibited colony formation (P < 0.0001 compared with vehicle). However, the combined treatment had markedly greater inhibitory effects than either drug alone in HCC1419 cells (P < 0.0001 compared with trastuzumab monotherapy, P < 0.05 compared with AMD3100 monotherapy; Fig. 1F and G) and HCC202 cells (both P < 0.0001 compared with each monotherapy; Fig. 1H and I). These results suggest that CXCR4 contributes to primary resistance to trastuzumab, and inhibition of CXCR4 sensitizes the cells to trastuzumab.

### Knockdown of CXCR4 abrogates trastuzumab resistance in HER2+ breast cancer cells

**Figure 2:**
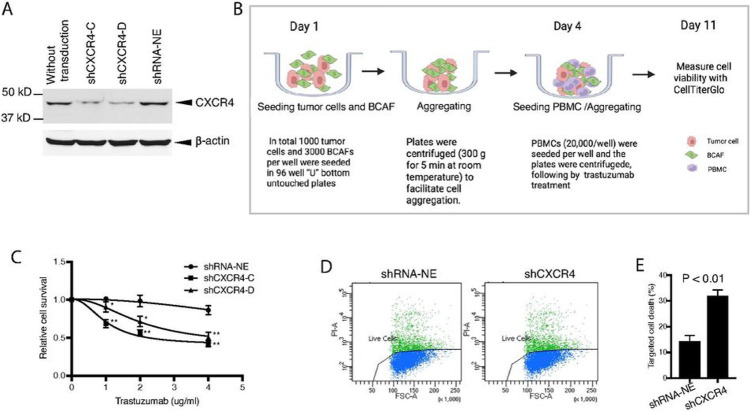
Knockdown of CXCR4 abrogates trastuzumab resistance. CXCR4 was silenced by specific shRNA in HCC1419 cells (Materials and Methods). The puromycin-resistant stable colonies were pooled together and named shCXCR4. A pool of cells infected with the lentivirus containing a non-effective vector (shRNA-NE) was selected and used as the control. **A** Western blot analysis was used to confirm the reduction in CXCR4 expression. **B, C** CXCR4-knockdown cells or non-silent control cells were co-cultured with BCAFs and PBMCs in 3D, followed by treatment with trastuzumab as illustrated (**B**). At the endpoint of the study, relative cell viability was quantitatively analyzed using CellTiter-Glo 3D viability assay kit, and the data were analyzed with one-way ANOVA using Prism (**C;** *P < 0.01, **P < 0.001 compared with the non-silent control cells). **D, E** CXCR4-knockdown cells or non-silent control cells were used for trastuzumab-induced antibody-dependent cellular cytotoxicity (detail in Materials and Methods). The cells were stained with propidium iodide and analyzed by flow cytometry (**D**). Data were analyzed using t-test analysis of variance and are reported as the mean ± SD of triplicates (**E**).

To further confirm the contribution of CXCR4 to trastuzumab resistance, we silenced CXCR4 using specific shRNA in HCC1419 cells with primary trastuzumab resistance (see Materials and Methods for details). Reduction of CXCR4 expression in the puromycin-resistant stable cell lines was confirmed (Fig. 2A). Because the effects of trastuzumab have been observed not only in tumor cells but also in tumor-host cells, specifically the recruitment of immune effector cells via their Fc domain [43,44], to mimic the tumor microenvironment, we co-cultured the tumor cells with or without CXCR4-knockdown as the target cells, BCAFs that produce SDF-1α, and PBMCs as the effector cells in 96-well “U” bottom unattached plates (Corning Life Science, NY). The spheres consisted of tumor cells, BCAFs, and PBMCs were treated with trastuzumab as illustrated in Fig. 2B. Cell viability was quantitatively analyzed (see Materials and Methods). Knockdown of CXCR4 significantly sensitized the tumor cells to trastuzumab (Fig. 2C).

We also performed trastuzumab-induced ADCC assays [37] (Materials and Methods. in detail). The HCC1419-derived cells were used as target cells, and the PBMCs were used as the effector cells. Consistent with the three-line co-culture above, flow cytometry analysis showed that CXCR4-knockdown cells exhibited an augmented response to trastuzumab (P < 0.01; Fig. 2D and E).

Taken together, these findings showed that CXCR4 plays a role in primary resistance to trastuzumab in HER2+ breast cancer, and combined targeting of CXCR4 sensitizes the tumor cells to trastuzumab.

### Continuous trastuzumab challenge induces acquired drug resistance and upregulation of CXCR4

**Figure 3:**
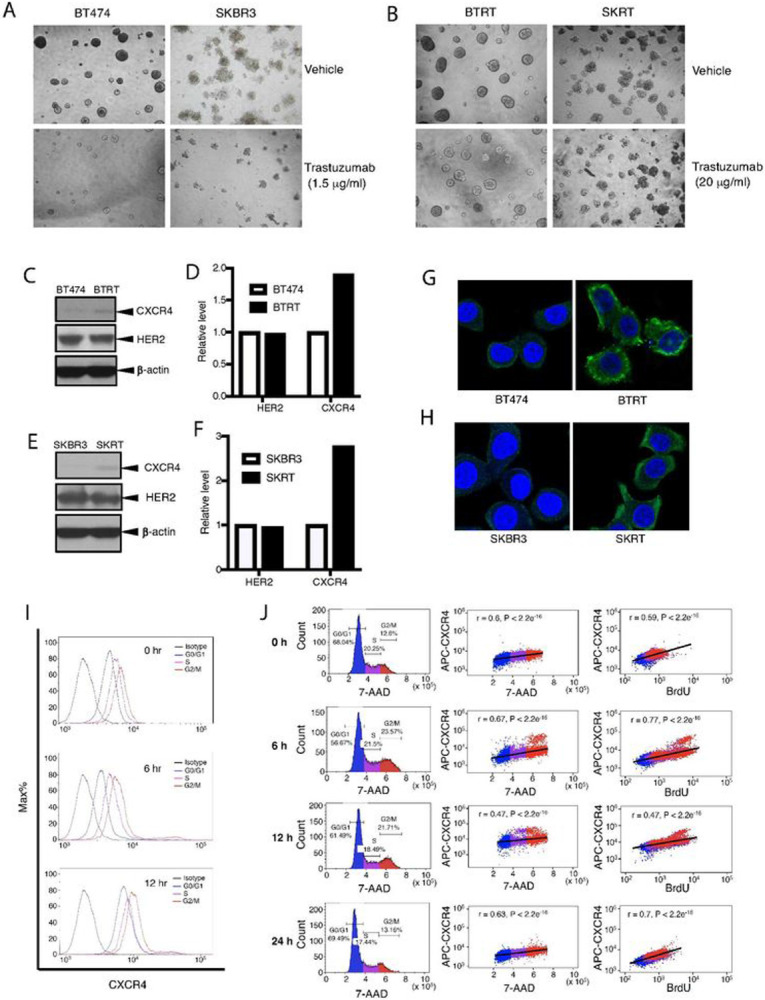
Creation of acquired trastuzumab-resistant cell models and characteristics of CXCR4 expression. To create cell models of acquired trastuzumab resistance, we continuously exposed BT474 and SKBR3 cells to trastuzumab (20 μg/ml) for at least 1 year. Tolerances to trastuzumab of primary cells (**A**) or the trastuzumab-resistant cells (**B**) were tested in 3D Matrigel culture. Photographs were taken on day 13. Expression of CXCR4 and HER2 was evaluated in BTRT (**C**) and SKRT (**E**) cells. Quantitative analysis of the density was performed using AlphaView SA software (**D, F**). CXCR4 expression in BTRT (**G**) and SKRT (**H**) cells was verified with immunofluorescent staining (green) and examined under a confocal microscope (see Materials and Methods). **I** Dynamic expression of CXCR4 with cell cycle progression in BTRT cells was detected by flow cytometry. **J**BTRT cells received BrdU pulse. Immunofluorescent staining for CXCR4, BrdU, and 7-AAD was performed and followed by flow cytometry analysis (Materials and Methods). The correlation between CXCR4 and 7-AAD or BrdU was analyzed using Pearson r coefficients.

To confirm that CXCR4 plays a role in acquired trastuzumab resistance, we created trastuzumab-resistant breast cancer models via continuous exposure of the trastuzumab-sensitive cells to trastuzumab (20 μg/ml) for at least 1 year. BT474 and SKBR3 cell lines were used to represent HER2+/estrogen receptor (ER)+ and HER2+/ER− breast cancer, respectively. The cells that acquired trastuzumab resistance were designated as BTRT and SKRT, respectively. Drug resistance was verified in the cells. Trastuzumab at a low concentration (1.5 μg/ml) markedly inhibited the primary cell growth in 3D Matrigel culture (Fig. 3A). As expected, the cells with acquired trastuzumab resistance exhibited tolerance to trastuzumab at much higher concentration (20 μg/ml; Fig. 3B). Upregulation of CXCR4 protein was found in both BTRT cells (Fig. 3C and D) and SKRT cells (Fig. 3E and F) compared with BT474 and SKBR3 cells, respectively, whereas HER2 expression did not change significantly after acquired trastuzumab resistance. Consistent with the Western blot analysis results, immunofluorescence staining showed overexpression of CXCR4 in BTRT (Fig. 3G) and SKRT cells (Fig. 3H). These results indicate that CXCR4 upregulation is associated with acquired trastuzumab resistance.

### CXCR4 expression increases with cell cycle progression and reaches a peak in the G2/M phases

We next investigated the dynamic expression of CXCR4 in acquired trastuzumab-resistant cells with the BrdU assay, in which BrdU was incorporated into newly synthesized DNA and stained with the FITC-conjugated anti-BrdU antibody; total DNA was detected with 7-amino-actinomycin D (7-AAD) and a specific primary antibody for CXCR4 and an APC-conjugated secondary antibody were used to detect CXCR4 (Materials and Methods in detail). Three-color flow cytometry analysis permits testing CXCR4 expression in different phases of the cell cycle. CXCR4 expression steadily increased from G0/G1 phase to S phase and reached the highest level in the G2/M phases (Fig. 3I). Pearson correlation coefficient analysis showed a high positive coefficient between CXCR4 expression and total DNA content, the two continuous variables (Fig. 3J, middle panel). Results at 6 hours and 12 hours after BrdU pulse showed higher CXCR4 expression in newly divided BrdU-positive cells than in relatively aged BrdU-negative cells, but 24 hours later, CXCR4 expression returned to baseline (Fig. 3J, right panel). The dynamic variation of CXCR4 supports that CXCR4 expression is associated with cell cycle progression in trastuzumab-resistant breast cancer cells.

### Inhibition of CXCR4 reverses the aggressive behavior of breast cancer cells with acquired trastuzumab resistance

**Figure 4:**
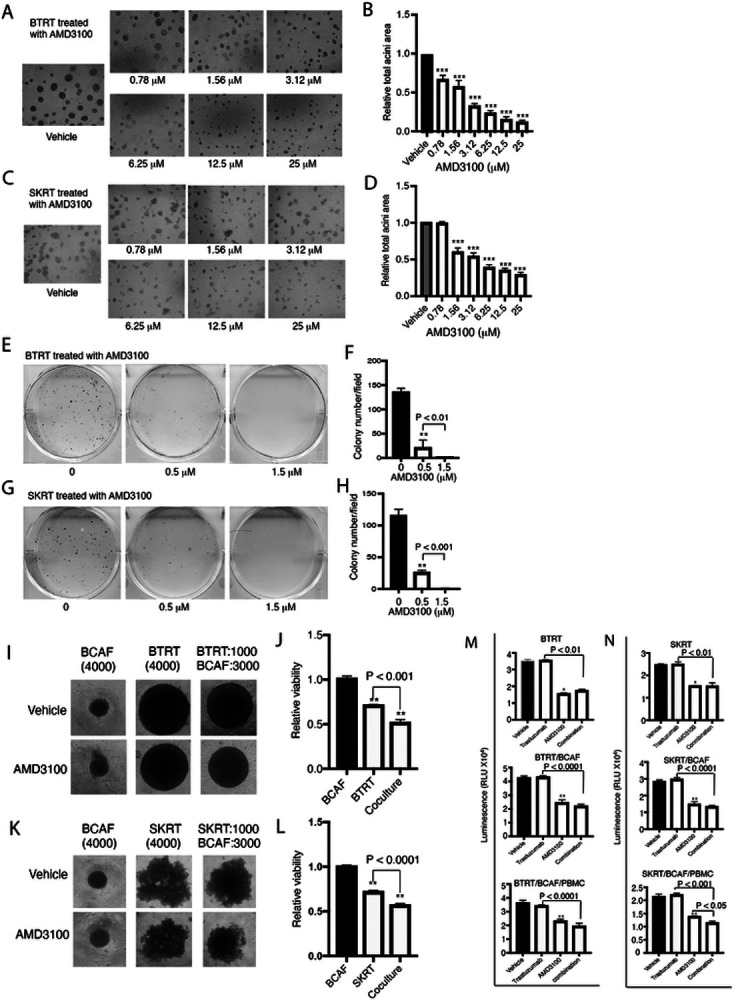
CXCR4 antagonist inhibits aggressive behavior in HER2+ breast cancer cells with acquired trastuzumab resistance. BTRT (**A**) and SKRT (**C**) cells were grown in 3D Matrigel followed by treatment with serial doses of AMD3100 (Materials and Methods). Photographs were taken on day 13. The total acini area was quantitatively analyzed with AlphaView SA. The data were analyzed using one-way ANOVA (**B, D**). BTRT (**E**) and SKRT (**G**) cells were seeded at low density and treated with different doses of AMD3100. The plates were scanned on day 18. Colony numbers were quantitatively analyzed using AlphaView SA. The data were analyzed using one-way ANOVA (**F, H**). BTRT (**I**) and SKRT (**K**) cells were co-cultured with BCAFs in 96-well “U” bottom unattached plates and treated with AMD3100 (2.5μM; Materials and Methods). Dynamic changes of the spheres were monitored and photographed. At the end of the study, viability of the cells in monoculture or co-culture was detected using CellTiter-Glo 3D viability assay kit. The cell viability ratio of treated with AMD3100 to vehicle was analyzed using one-way ANOVA (**J, L**). BTRT (**M**) and SKRT (**N**) cells were co-cultured with BCAFs (two lines) or with BCAFs and PBMCs (three lines), followed by treatment with trastuzumab (20 μg/ml) and/or AMD3100 (2.5μM) as illustrated in Fig S1. At the endpoint, cell viability was detected using CellTiter-Glo 3D viability assay kit and analyzed using one-way ANOVA. The data are reported as mean ± SD of triplicates, representing two independent experiments (*P < 0.01, **P < 0.001, ***P < 0.0001 compared with vehicle).

To investigate whether targeting the cell cycle progression-associated CXCR4 affects cell proliferation, we seeded BTRT and SKRT cells in Matrigel and treated the cells with AMD3100. AMD3100 dose-dependently inhibited acini growth of BTRT (Fig. 4A and B) and SKRT (Fig. 4C and D) cells (P < 0.0001 compared with vehicle). We also tested the effect of AMD3100 on cell survival using clonogenic assays. With a similar pattern to that exhibited in cell growth assays, AMD3100 dose-dependently inhibited colony formation in BTRT (Fig. 4E and F) and SKRT (Fig. 4G and H) cells (P < 0.0001 compared with vehicle).

To mimic the microenvironment of breast cancer, we again co-cultured trastuzumab-resistant HER2+ breast cancer cells with BCAFs followed by treatment with or without AMD3100. The monocultures were used as controls. Spheres were photographed every 4 days. Compared with vehicle, AMD3100 inhibited growth of the spheres formed by BTRT cells, but not those formed by BCAFs. However, the inhibitory effect was further increased in the co-culture of BTRT and BCAFs (Fig. 4I; Fig. S2). Co-cultures of SKRT with BCAFs showed similar results (Fig. 4K; Fig. S2). At the end of the study, cell viability was quantitatively analyzed (Materials and Methods in detail). Consistent with the size of spheres, AMD3100 inhibited the viability of BTRT and SKRT cells in monoculture (P < 0.0001). The inhibitory effect was further increased in the co-cultures of BTRT cells and BCAFs (P < 0.001; Fig. 4J) and SKRT cells and BCAFs (P < 0.0001; Fig. 4L) but did not affect the viability of BCAFs compared with vehicle.

Because growing evidence suggests that trastuzumab requires the engagement of the immune system for effectiveness [43, 44], we further co-cultured the tumor cells with BCAFs and PBMCs, and then treated the spheres with AMD3100, trastuzumab, or the combination (Fig. S1). AMD3100 inhibited tumor cell growth in monoculture (P < 0.001) and co-culture (P < 0.0001). Adding trastuzumab to AMD3100 did not further increase the efficacy in BTRT monoculture, but mildly increased the inhibitory efficacy in cocultures, particularly with immune engagement (Fig. 4M). As expected, trastuzumab alone did not inhibit viability of the tumor cells with acquired trastuzumab resistance in monoculture or co-cultures with BCAFs and/or PBMCs. A similar pattern was observed in SKRT cells (Fig. 4N).

Taken together, these results indicate that CXCR4 contributes to acquired trastuzumab resistance, and targeting CXCR4 with its antagonist reverses resistance.

### Targeting CXCR4 with AMD3100 restrains cell division by inhibiting mediators of G2-M transition and mitosis

**Figure 5:**
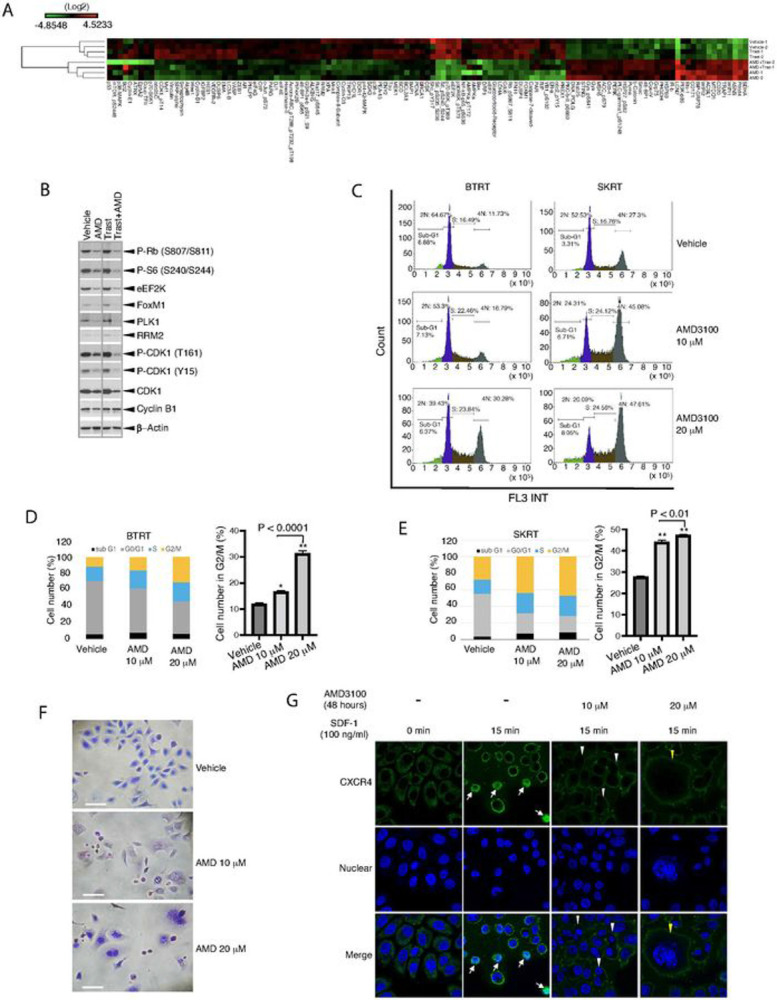
Mechanism of AMD3100 function in HER2+ breast cancer with trastuzumab resistance. **A** BTRT cells grown in 3D Matrigel culture were treated with vehicle, AMD3100 (10μM), trastuzumab (20 μg/ml), or their combination. Cell lysates were subject to RPPA (Materials and Methods). Data are presented in a matrix format: each column represents an antibody target and each row a sample. In each sample, the ratio of the abundance of the molecule to its median abundance across all samples is represented by the color of the corresponding cell in the matrix (see the scale for expression levels). **B** Western blot analysis. **C** BTRT and SKRT cells grown in 3D Matrigel culture were treated with AMD3100 for 3 days. The cells were collected from the Matrigel and analyzed to determine the phases of the cell cycle using flow cytometry. **D, E** Quantitative analysis was performed, and the data were analyzed using one-way ANOVA. The data are reported as mean ± SD of triplicates, representing two independent experiments (*P < 0.001, **P < 0.0001 compared with vehicle). **F** SKRT cells grown on coverslips pre-coated with poly-L-lysine were treated with AMD3100 or vehicle in the growth medium for 72 hours. The cells were stained with the Kwik Diff Stains kit (Scale bar, 50 μm). **G**SKRT cells were similarly grown on coverslips and treated with AMD3100 or vehicle in the growth medium for 48 hours. After serum starvation overnight, the cells received SDF-1a (100 ng/ml) stimulation for the times indicated and were fixed and permeabilized. CXCR4 was detected with mouse anti-human CXCR4 primary antibody (R&D, Minneapolis, MN) and the Alexa Fluor 488–conjugated goat anti-mouse secondary antibody (green). Nuclei were stained with DAPI (blue). Microscopic images were captured by a multiphoton confocal laser-scanning microscope (Materials and Methods). Arrows indicate CXCR4 nuclear translocation, white triangles indicate binucleated cells, and yellow triangles indicate giant multinucleated cells.

Our studies above demonstrated that the CXCR4 antagonist AMD3100 inhibits proliferation and survival of HER2+ breast cancer cells with primary or acquired trastuzumab resistance. To further discern the mechanism of these effects, we performed functional proteomic analyses. BTRT cells grown in Matrigel 3D culture were treated with vehicle, AMD3100, and/or trastuzumab. Cell lysis was analyzed using RPPA with 484 antibodies (Table S1). Unsupervised hierarchical clustering showed that AMD3100 monotherapy and combined therapy with trastuzumab formed a cluster at the bottom of the dendrogram (Fig. S3). As expected, trastuzumab monotherapy did not result in a distinct cluster but formed a cluster with the vehicle, likely because cells had adapted to continuous exposure to trastuzumab. Fig. 5A is an enlarged image of the left part of the panel, showing the significant difference between the two main clusters.

As expected, targeting CXCR4 with AMD3100 inhibited downstream signaling pathways of the G protein–coupled receptor, including the MAPK pathway, as indicated by decreased levels of phosphorylation of ERK1/2, p90RSK, p70RSK, S6, and c-Jun, and the PI3K-AKT-mTOR pathway, as shown by decreased phosphorylation of NF-κB, GSK3α/ , mTOR, 4EBP1, YB-1, and Rb. AMD3100 also reduced the molecules that we demonstrated upregulation in the trastuzumab-resistant breast cancer cells comparing their parental cells, including ERa, Notch3, IGFBP2, and dual specificity phosphatase 4 (DUSP4), which contribute to cancer formation and progression or resistance to anti-HER2 therapy or chemotherapy [45-47]. Intriguingly, AMD3100 suppressed many regulators of the G2/M phases of the cell cycle, particularly, those involved in the G2-M transition checkpoints, as indicated by downregulation of cyclin B1, Wee1, Myt1, CDC25C, FoxM1, eEF2K, and reduced the phosphorylation of CDK1, Rb, 4EBP-1and S6 (Fig. 5A, Fig. S4). The RPPA data were confirmed with Western blot analysis (Fig. 5B, Fig. S5). The results suggest that AMD3100 functions at the CXCR4-high expression G2/M phases.

The results from molecular analysis led us to investigate whether targeting CXCR4 affects cell division. Cell cycle analysis showed that AMD3100 dose-dependently increased the number of cells in the G2/M phases in BTRT (Fig 5C and D) and SKRT cells (Fig 5C and E). When the AMD3100-treated SKRT cells were analyzed using flow cytometry, a group of cells was automatically identified as doublets, which led us to examine the cell morphology using a modified Wright-Giemsa stain. As expected, AMD3100 induced significant morphologic changes, as indicated by binucleated or giant multinucleated cells (Fig. 5F), which were very likely identified as doublets by flow cytometry or were filtered before upload.

We next verified the function of AMD3100 using fluorescence confocal microscopy. SKRT cells were treated with or without AMD3100 and followed by stimulation with SDF-1α. In regular culture, without treatment and stimulation, CXCR4 is mainly located in the cytoplasm of the cells. After stimulation with SDF-1α for 15 minutes, cytoplasmic CXCR4 was reduced, and membrane-associated CXCR4 was increased. The cells became smaller, and some of them exhibited translocation of CXCR4 into the nuclei (Fig. 5G). The changes in CXCR4 and cell size returned to normal in 30 minutes (Fig. S6). As expected, AMD3100 dose-dependently induced obvious morphologic changes, with binucleated and giant multinucleated cells, and inhibited CXCR4 nuclear translocation (Fig. 5G, right panels). Treatment with AMD3100 for 72 hours did not induce apoptosis in either BTRT (Fig. S7A) or SKRT cells (Fig. S7B).

Taken together, these results showed that targeting of CXCR4 with AMD3100 arrests cell division by inhibition of the mediators of G2-M transition and mitosis but does not induce apoptosis.

### Combined targeting CXCR4 and docetaxel synergistically inhibits trastuzumab resistant tumor cell growth in vitro and significantly improves the inhibitory efficacy in vivo

**Figure 6:**
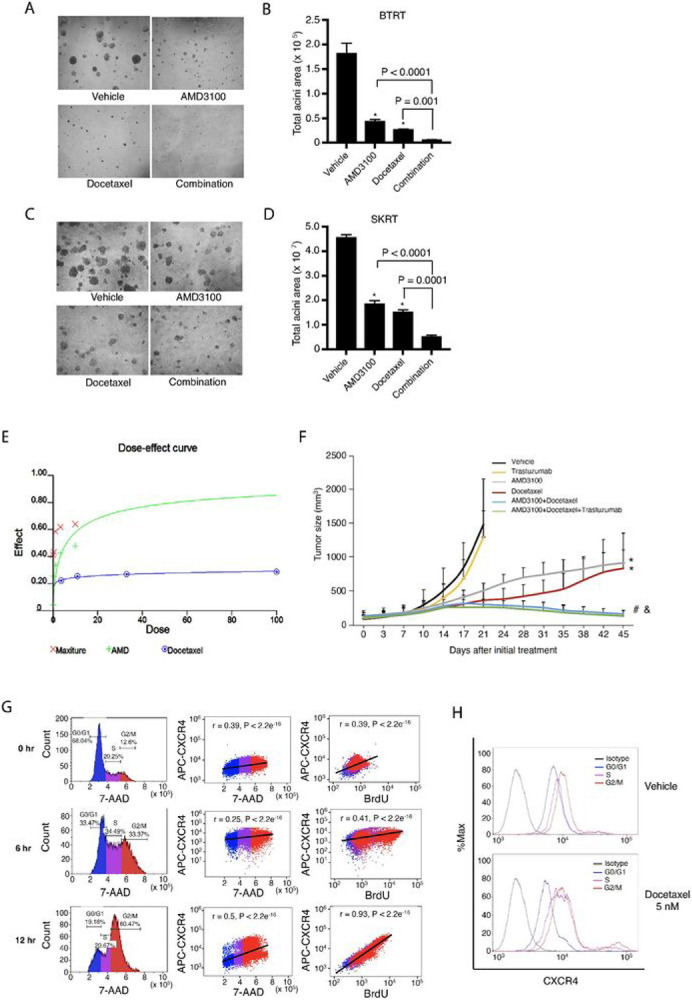
Effect of combined treatment with AMD3100 and docetaxel on acquired trastuzumab-resistant breast tumor growth. **A, C** BTRT or SKRT cells were grown in 3D Matrigel culture and treated with AMD3100 (5 μM), docetaxel (10 nM), or the combination. Photographs were taken on day 9. **B, D** Quantitative analysis of total acini area was performed using AlphaView SA, and the data were analyzed using one-way ANOVA. The data are reported as mean ± SD of triplicates, representing two independent experiments (*P < 0.0001 compared with vehicle). **E** BTRT cells grown in 3D Matrigel were treated with serial doses of AMD3100 (AMD) and/or serial doses of docetaxel. The combined effect of AMD3100 and docetaxel on acini growth was analyzed using CalcuSyn Dose Effect Analyzer. **F** HR6 cells, derived from BT474 cells and exhibiting in vivo acquired trastuzumab resistance, were implanted into the mammary fat pad of female athymic nude mice. When the tumor size reached 100 mm, the mice were randomized to treatment with vehicle, trastuzumab, AMD3100, docetaxel, or different combinations. Tumor volume was calculated using the formula V = lw^2^/2. Data were analyzed using two-way ANOVA (*P < 0.0001 compared with vehicle, ^#^P < 0.0001 compared with AMD3100 alone, ^&^ P < 0.0001 compared with docetaxel alone). **G** BTRT cells were treated with docetaxel (5 nM) followed by BrdU pulse. Immunofluorescent staining for CXCR4, BrdU, and 7-AAD was performed and followed by flow cytometry analysis (Materials and Methods). The correlation between CXCR4 and 7-AAD or BrdU was analyzed using Pearson r coefficients. **H** BTRT cells were treated with docetaxel or vehicle. Dynamic expression of CXCR4 with cell cycle progression in BTRT cells was detected by flow cytometry.

AMD3100 prolonged the cell cycle and slowed down cell growth but did not completely block the G2/M phases. Clinically, chemotherapy is a fundamental component of combined therapies for advanced HER2+ breast cancer except as maintenance following induction therapy [48]. To investigate whether adding CXCR4 inhibitor to chemotherapy improves efficacy, and which chemotherapy reagents produce the best combinatorial effect, we tested the combination of AMD3100 with cisplatin, carboplatin, and docetaxel. Treatment with AMD3100 or docetaxel inhibited BTRT cell growth in 3D Matrigel culture (P < 001 compared with vehicle). However, the combination of AMD3100 and docetaxel significantly increased the inhibitory effects compared with either drug alone, as indicated by almost completely inhibited acini growth (P < 0.0001 compared with AMD3100 alone, P < 0.001 compared with docetaxel alone; Fig. 6A and B). The inhibitory effects exhibited a similar pattern in SKRT cells (Fig. 6C and 6D). We next treated BTRT cells with serial doses of AMD3100 (AMD) and/or docetaxel, followed by synergy analyses. The dose-effect curve (Fig. 6E) and combination indices (Table 1) from the synergy analyses indicated synergistic interactions. However, combination of cisplatin and AMD3100 did not increase their inhibitory effects on cell growth of BTRT (Fig. S8A and S8B) or SKRT (Fig. S8C and S8D) in Matrigel, even had the opposite effect. The findings were recapitulated using carboplatin to replace cisplatin on BTRT cells (Fig. S8E and S8F) SKRT cells (Fig. S8G and S8H)

To verify our findings in vivo, we used HR6, an acquired trastuzumab resistant xenograft model, which were derived from BT474 cells and created by trastuzumab challenge in athymic nude mice [12]. To confirm trastuzumab resistance, we transplanted the HR6 cells into the mammary fat pad of athymic nude mice that have natural killer cells and macrophages/monocytes; these mice are capable of generating antibody-dependent cellular cytotoxicity even though they lack T cells. BT-T cells [12, 49], derived from parental BT474 cells and remaining sensitive to trastuzumab in athymic nude mice, were used as a control. After the tumor size reached 100 mm, all mice were treated with trastuzumab (Materials and Methods in detail). As expected, trastuzumab inhibited xenograft growth of BT-T but not HR6 (Fig. S9). We next established HR6 xenografts using the same method. The mice with tumor burden were randomly assigned to treatment with vehicle, trastuzumab, AMD3100, docetaxel, or different combinations (Fig. 6F). As expected, trastuzumab did not show an inhibitory effect. AMD3100 or docetaxel monotherapy significantly inhibited the growth of the xenografts (P < 0.0001 compared with vehicle). However, the combination of AMD3100 and docetaxel further induced tumor regression (P < 0.0001 compared with AMD3100 or docetaxel alone). The addition of trastuzumab to AMD3100/docetaxel tended to increase the inhibitory effect, but the difference was not significant, suggesting that after long-term exposure to trastuzumab, the tumor cells adapted to the drug.

Taken together, these results indicated that combined targeting CXCR4 with AMD3100 and docetaxel is a potential novel combination therapy for HER2+ breast cancer with trastuzumab resistance.

### AMD3100 synergistically interacts with docetaxel by suppressing docetaxel-induced CXCR4 upregulation in trastuzumab-resistant breast cancer

We next explored the mechanism of the synergistic interactions of AMD3100 and docetaxel. After being treated with docetaxel, BTRT cells received BrdU pulse, and then dynamic expression of CXCR4 in the cell cycle phases was measured using flow cytometry (Materials and Methods; Fig. 3J). As expected, the microtubule inhibitor docetaxel arrested the cells in the M phase (Fig. 6G, left panel). CXCR4 expression levels markedly increased from the S phase and reached a peak in the G2/M phases (Fig. 6G and 6H). CXCR4 protein levels reached their highest point at 12 hours after treatment and were highly correlated with BrdU (r = 0.93, P < 2.2e^−18^; Fig. 6G, right panel). These results indicate that CXCR4 upregulation is a response of the cells to docetaxel, possibly a self-protective mechanism. The addition of AMD3100 suppressed the response to docetaxel, thus synergistically inhibiting tumor cell growth.

### CXCR4 is upregulated in residual diseases than primary breast tumors

**Figure 7:**
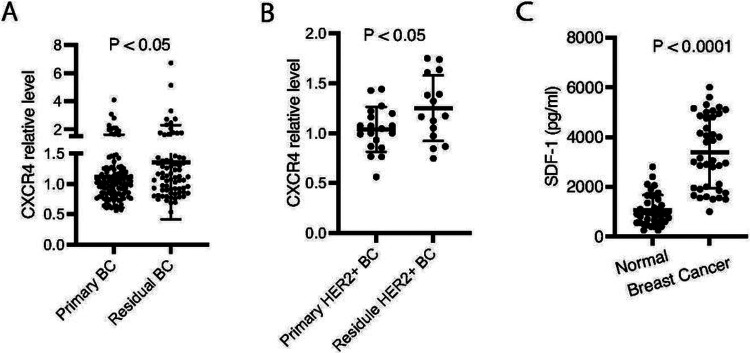
Upregulation of CXCR4 in residual disease of breast cancer. **A** CXCR4 protein in 72 residual tumor tissues and 112 primary tumor samples from breast cancer patients was analyzed using RPPA. **B** CXCR4 protein in residual HER2+ breast cancer after treatment with trastuzumab and chemotherapy compared with primary HER2+ breast cancer. **C** Serum samples from breast cancer patients or healthy women was measured for SDF-1α with ELISA. Data were analyzed using t-test.

To investigate the role of CXCR4 in trastuzumab resistance in breast cancer patients, we performed a retrospective study, in which CXCR4 expression in fresh-frozen tumor tissues from 72 patients who received neoadjuvant before surgery (residual tumor tissues) and 112 untreated patients (primary breast tumor tissues) was tested using RPPA. CXCR4 expression was increased in the residual disease samples compared with the primary tumors (P < 0.05; Fig. 7A). In the cohort tested, in total 34 samples were HER2+, including 19 primary tumor tissues and 15 residual tumor samples. Compared with the primary tumor tissues, the residual disease samples exhibited higher CXCR4 protein after treatment with trastuzumab and chemotherapy (P < 0.05) (Fig. 7B). Taken together, the evidence supports the contribution of CXCR4 to drug resistance. We also measured SDF-1a in serum using ELISA. Circulating SDF-1a levels were significantly higher in blood samples from breast cancer patients comparing the healthy controls (P < 0.0001) (Fig. 7C).

## Discussion

We previously showed upregulation of CXCR4 is involved as a driver of trastuzumab resistance in HER2+ breast cancer cells [18-20]. In the current study, we demonstrated that the increased CXCR4 expression is associated with cell cycle progression and reaches a peak in the G2/M phases. To our knowledge, this is the first study to demonstrate that CXCR4 plays a role in cell cycle progression in cancer cells, although a similar phenomenon was reported in germinal center B cells [50]. Our functional proteomic analysis showed that targeting CXCR4 with its antagonist AMD3100 downregulated the G2-M transition–associated proteins that are strictly required to complete mitosis. Consistently, flow cytometry analysis indicated G2-M arrest, and imaging analysis showed multinucleation and abnormal mitosis. The molecular changes and cellular biological phenomena induced by AMD3100 and the characteristics of CXCR4's dynamic expression with cell cycle progression corroborate each other.

Using functional proteomics analysis, we examined CXCR4 protein expression in 184 fresh-frozen breast tumor tissues, including 34 HER2+, from breast cancer patients with or without chemotherapy and/or trastuzumab treatment, consistent with our findings from the cell lines, comparing the primary tumors, CXCR4 expression significantly increased in the residual tumor tissues, suggesting that CXCR4 can be a biomarker to predict the drug resistance. Supporting our findings, in a very recent report, a retrospective clinical study investigated CXCR4 expression in 62 formalin-fixed paraffin-embedded tissue specimens using RT-qPCR and immunohistochemistry and found upregulation of CXCR4 in trastuzumab-treated samples. High CXCR4 expression was associated with recurrence [21]. Teams from different countries using different methods showed CXCR4 upregulation in breast cancer with trastuzumab resistance.

Breast cancer cells cultured in 3D showed different responses to chemotherapies than those observed in cells cultured in 2D [51]. Given the known role of CXCR4 signaling in the tumor/immune microenvironment, to better understand how CXCR4 signaling contributes to trastuzumab resistance, we used 3D co-culture (tumor cells/ breast cancer-associated fibroblasts/ human peripheral blood mononuclear cells) or 3D Matrigel culture with the supplement of SDF-1a, referencing its concentration in breast cancer patients. Recapitulating the stromal and immune environment in our model systems demonstrated an enhanced impact of our strategy as expected.

Studies showed that CXCR4 is involved in resistance to chemotherapies, including paclitaxel, a taxane similar to docetaxel, in ovarian cancer [52] and ER+ or triple-negative breast cancer cell lines [53]. Our study, as the first, demonstrated that targeting CXCR4 synergizes with docetaxel in HER2+ breast cancer with trastuzumab resistance. We also identified the direct mechanism that CXCR4 upregulation is a response of the tumor cells to docetaxel, and AMD3100 blocks the protective adaptation.

## Conclusion

The present study provided preclinical evidence that targeting CXCR4 abrogates trastuzumab resistance by blocking cell cycle progression and synergizes with docetaxel in trastuzumab-resistant breast cancer treatment. Our findings therefore demonstrated that CXCR4 is a promising therapeutic target and a predictive biomarker in HER2 + breast cancer with trastuzumab resistance. Our next goal is a biomarker-driven prospective trial of trastuzumab plus CXCR4 inhibitor either with or without docetaxel in patients who have exhibited resistance to trastuzumab.

## Abbreviations

HER2+: Human epidermal growth factor receptor 2 positive
PI3K: Phosphatidylinositide 3 kinase
IGF-1R: The insulin-like growth factor-I receptor
EGFR: Epidermal growth factor receptor
CXCR4: C-X-C motif chemokine receptor 4
CXCL12: C-X-C motif chemokine ligand 12
SDF-1: Stromal cell-derived factor-1
3D: Three-dimensional
BrdU: 5'-bromo-2'-deoxyuridine
FBS: fetal bovine serum
7-AAD: 7-amino-actinomycin D
DAPI: 4’, 6-diamidino-2-phenylindole
ELISA: Enzyme-linked immunosorbent assay
CFDA, SE: 5- (and 6)-carboxyfluorescein diacetate, succinimidyl ester
EDTA: Ethylenediaminetetraacetic acid
ADCC: Antibody-dependent cellular cytotoxicity
MAPK: Mitogen-activated protein kinase
PBMC: Peripheral blood mononuclear cell
ER: Estrogen receptor
BCAF: Breast cancer-associated fibroblast
RPPA: Reverse phase protein array
ANOVA: Analysis of variance
PBS: Phosphate-buffered saline
GPCR: G-protein coupled receptor
ER+: Estrogen Receptor positive
ELISA: Enzyme-linked immunosorbent assay
BTRT: BT474-derived trastuzumab-resistant cells
SKRT: SKBR3-derived trastuzumab-resistant cells
